# Draft Genome Sequences of 18 *Salmonella enterica* subsp. *enterica* Serovar Oranienburg Strains Isolated from Rivers in Northwestern Mexico

**DOI:** 10.1128/genomeA.01585-16

**Published:** 2017-03-09

**Authors:** Gloria M. Casteñeda-Ruelas, César Carreón-Gaxiola, Hugo G. Castelán-Sánchez, Abraham Acatzi-Silva, Salvador Romero-Martínez, Alejandra García-Molina, Maribel Jiménez-Edeza

**Affiliations:** a Laboratorio de Investigación y Diagnóstico Microbiológico (LIDiM), Programa Regional en Biotecnología, Universidad Autónoma de Sinaloa, Culiacan, Sinaloa, Mexico; b Centro Nacional de Referencia en Detección de Organismos Genéticamente Modificados (SENASICA), Tecámac, Estado de Mexico, Mexico

## Abstract

*Salmonella enterica* subsp. *enterica* serovar Oranienburg is recognized as a foodborne pathogen widely distributed in the environment. Here, we report 18 draft genomes of *S*. Oranienburg strains isolated from rivers in the northwestern region of Mexico.

## GENOME ANNOUNCEMENT

In Mexico, the Dirección General de Epidemiología (General Directorate of Epidemiology), highlights *Salmonella* as one of the main etiological agents of gastroenteritis, and reports 72,000 nontyphoid salmonellosis (NTS) cases annually (http://www.epidemiologia.salud.gob.mx/anuario/html/anuarios.html). However, it should be noted that serotypes linked to these cases and the infection route were not clearly defined. *Salmonella enterica* subsp. *enterica* serovar Oranienburg is a nontyphoid serotype associated with foodborne outbreaks worldwide, including México (1). The rivers located in the northwestern region of Mexico have been recognized as a harboring source of different clones of *S*. Oranienburg strains and are irrigation sources of crop-exported production, exposing the potential risk of *Salmonella* dissemination (2). Irrigation water used in agriculture has been linked to NTS outbreaks in the United States (3). Hence, we report here the availability of draft-genome sequences of 18 *S*. Oranienburg strains isolated from diverse river points in northwestern Mexico.

The bacterial strains samples were prepared by a modified High Pure PCR template preparation kit (Roche, Indianapolis, IN) that carried out genomic DNA extraction. Following the manufacturer’s protocol, sequencing libraries were prepared using the Nextera XT kit (2 × 251 bp) (Illumina, San Diego, CA). Whole genomes were sequenced on an Illumina MiSeq platform (Illumina, San Diego, CA). PEAT-V1-2.1.4 (4) and Trimmomatic version 0.36 (5) were used for adapter and quality trimming, respectively. Assembling analysis was performed with the pipeline A5-miseq (6), and the Rapid Annotations using Subsystems Technology (RAST) server (http://rast.nmpdr.org/) and NCBI Prokaryotic Genomes Automatic Annotation Pipeline (http://www.ncbi.nlm.nih.gov/genome/annotation_prok) were used to annotate the assembled genomes. Genome size and G+C content were estimated with all contigs of each strain. Also, the serotype was determined by SeqSero software (7).

The *in silico* serotyping confirmed that all of the strains belong to the *S*. Oranienburg (7:m,t:-) serotype, as previously reported by a conventional serological test (2). Table 1 summarizes the general characteristics of the draft-genomes of the 18 *S*. Oranienburg strains isolated from rivers in northwestern Mexico. Briefly, the draft genomes among the strains consist of ~4.57 to ~5.00 Mb with a median value for G+C of 52.0%, and on average a total of ~4,714 and 4,591 coding DNA sequences and proteins were identified, respectively (Table 1). At least 2.2% of proteins might be related to virulence functions, which could be used to infer phylogenetic relationships between strains. These genome sequences have been deposited in GenBank, contributing to the number of *S*. Oranienburg genomes and biological evolutionary knowledge. Additionally, these references can be used to contribute to epidemiological surveillance studies to control this foodborne pathogen.

**TABLE 1:** Metadata for *Salmonella* Oranienburg strains isolated from river water in northwestern Mexico

GenBank accession no.	No. of contigs	Genome size (Mb)	G+C content (%)	No. of coding DNA sequences (bp)	No. of proteins	No. of virulence genes	Coverage (fold)
MEJA00000000	157	4.9	51.9	4,956	5,076	111	34
MEJJ00000000	38	4.6	52.1	4,495	4,610	108	87
MEJK00000000	40	4.6	52.1	4,497	4,614	109	45
MEJL00000000	40	4.6	52.1	4,493	4,610	107	38
MEJM00000000	89	4.9	51.9	4,932	5,054	109	57
MEJN00000000	42	4.6	52.1	4,503	4,620	104	36
MEJO00000000	51	4.6	52.1	4,493	4,612	108	38
MEJP00000000	113	5.0	52.1	5,090	5,201	102	59
MEJQ00000000	72	4.7	52.0	4,566	4,685	108	68
MEJR00000000	44	4.6	52.1	4,493	4,607	108	75
MEJS00000000	75	4.6	52.1	4,499	4,620	108	49
MEJT00000000	55	4.6	52.1	4,504	4,623	108	47
MEJU00000000	83	5.0	52.0	4,978	5,095	109	56
MEJV00000000	117	5.0	52.0	4,991	5,102	108	43
MEJW00000000	92	4.9	52.0	4,726	4,838	108	42
MEJX00000000	34	4.6	52.1	4,496	4,607	108	55
MEJY00000000	43	4.6	52.1	4,442	4,557	108	58
MEJZ00000000	110	5.0	51.9	4,946	5,052	109	31

### Accession number(s).

The genome sequences of these 18 *S*. Oranienburg strains are part of GenBank BioProject PRJNA186035 under the accession numbers listed in Table 1.
